# The Microglial Transcriptome of Age-Associated Deep Subcortical White Matter Lesions Suggests a Neuroprotective Response to Blood–Brain Barrier Dysfunction

**DOI:** 10.3390/ijms25084445

**Published:** 2024-04-18

**Authors:** Taghreed Almansouri, Rachel Waller, Stephen B. Wharton, Paul R. Heath, Fiona E. Matthews, Carol Brayne, Fredericus van Eeden, Julie E. Simpson

**Affiliations:** 1 Sheffield Institute for Translational Neuroscience, University of Sheffield, Sheffield S10 2HQ, UK; talmansouri@kau.edu.sa (T.A.); r.waller@sheffield.ac.uk (R.W.); s.wharton@sheffield.ac.uk (S.B.W.); p.heath@sheffield.ac.uk (P.R.H.); 2 Department of Medical Laboratory Technology, Faculty of Applied Medical Sciences, King Abdulaziz University, Jeddah 21589, Saudi Arabia; 3 Institute for Clinical and Applied Health Research, University of Hull, Hull HU6 7RX, UK; f.matthews@hull.ac.uk; 4 Department of Psychiatry, University of Cambridge, Cambridge CB2 3EG, UK; cb105@medschl.cam.ac.uk; 5 School of Biosciences, University of Sheffield, Sheffield S10 2GF, UK; f.j.vaneeden@sheffield.ac.uk

**Keywords:** deep subcortical lesions, microglia, transcriptomic profiling

## Abstract

Age-associated deep-subcortical white matter lesions (DSCLs) are an independent risk factor for dementia, displaying high levels of CD68^+^ microglia. This study aimed to characterize the transcriptomic profile of microglia in DSCLs and surrounding radiologically normal-appearing white matter (NAWM) compared to non-lesional control white matter. CD68^+^ microglia were isolated from white matter groups (*n* = 4 cases per group) from the Cognitive Function and Ageing Study neuropathology cohort using immuno-laser capture microdissection. Microarray gene expression profiling, but not RNA-sequencing, was found to be compatible with immuno-LCM-ed post-mortem material in the CFAS cohort and identified significantly differentially expressed genes (DEGs). Functional grouping and pathway analysis were assessed using the Database for Annotation Visualization and Integrated Discovery (DAVID) software, and immunohistochemistry was performed to validate gene expression changes at the protein level. Transcriptomic profiling of microglia in DSCLs compared to non-lesional control white matter identified 181 significant DEGs (93 upregulated and 88 downregulated). Functional clustering analysis in DAVID revealed dysregulation of haptoglobin–haemoglobin binding (Enrichment score 2.5, *p* = 0.017), confirmed using CD163 immunostaining, suggesting a neuroprotective microglial response to blood–brain barrier dysfunction in DSCLs. In NAWM versus control white matter, microglia exhibited 347 DEGs (209 upregulated, 138 downregulated), with significant dysregulation of protein de-ubiquitination (Enrichment score 5.14, *p* < 0.001), implying an inability to maintain protein homeostasis in NAWM that may contribute to lesion spread. These findings enhance understanding of microglial transcriptomic changes in ageing white matter pathology, highlighting a neuroprotective adaptation in DSCLs microglia and a potentially lesion-promoting phenotype in NAWM microglia.

## 1. Introduction

Age-associated white matter lesions (WMLs) appear as hyperintensities on T2-weighted magnetic resonance images (MRIs), which are broadly classified into periventricular lesions (PVLs) and deep subcortical lesions (DSCLs) [1]. The Cognitive Function and Ageing Study (CFAS) is a longitudinal study of dementia and frailty in an ageing population-representative cohort [2]. In the CFAS, PVLs have a prevalence of approximately 90% and DSCLs 60% in the ageing population [3]. These WMLs are associated with neurological diseases, such as Alzheimer’s disease [4,5,6] and are an independent predictor for cognitive impairment [7]. The exact mechanism of their formation remains unknown; however, several theories have been proposed including cerebral hypoperfusion, dysfunction of the blood–brain barrier (BBB) and neurodegeneration due to overlying cortical pathologies [3,8].

Central to the brain’s immune response, microglia play a pivotal role in maintaining homeostasis and responding to pathological changes [9]. Previous histological characterization of WMLs in CFAS has shown that while both DSCLs and PVLs are associated with extensive demyelination and astrogliosis, their microglial profile varies: DSCLs predominantly contain CD68^+^ microglia with an amoeboid morphology, while PVLs contain high levels MHC-II^+^ microglia with a ramified profile, as reviewed [10]. Microglia within the radiologically normal-appearing white matter (NAWM) from lesional cases express significantly elevated levels of MHC II compared to microglia from control non-lesional WM cases, and also display high levels of oxidative DNA damage and a DNA damage response [10], suggesting altered glial function extends beyond the WMLs and may contribute to lesion progression.

Transcriptomic profiling enables the gene expression profile of samples to be assessed, potentially identifying novel biologically relevant mechanisms associated with disease pathogenesis. Previous characterization of the microglial transcriptome in PVLs identified the PVLs as a continuous spectrum of WM injury [11]. Furthermore, the gene expression profile of DSCLs and surrounding NAWM suggests that alteration of cell metabolic pathways and glial cell injury contribute to DSCL pathogenesis [10]. However, this microarray approach analyzed mRNA extracted from the entire region of interest, which may have masked microglial specific gene expression changes in deep subcortical white matter. Therefore, to determine the microglial transcriptomic profile in DSCLs and identify gene expression changes which may contribute to age-associated white matter pathology, the current study performed gene expression profiling using both RNAseq and microarray analysis on an enriched population of laser capture microdissected microglia.

## 2. Results

### 2.1. Histological Characterization Confirms the Classification of Sampled WM

**Figure 1:**
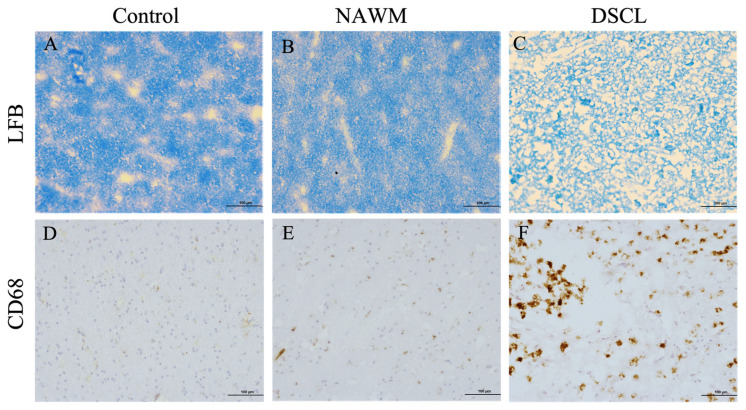
Histological characterization of deep subcortical white matter. Both non-lesional control white matter (**A**) and normal-appearing white matter (NAWM) (**B**) displayed a regular pattern of myelin staining and low levels of CD68^+^ ramified microglia (**D**,**E**). In contrast, age-associated deep subcortical lesions (DSCLs) displayed extensive demyelination (**C**) and high levels of CD68^+^ microglia with an amoeboid morphology (**F**). LFB: luxol fast blue. Scale bar represents 100 µm.

Both control WM from non-lesional cases and NAWM from lesional cases displayed a regular pattern of myelin staining (Figure 1A,B) and contained low levels of ramified microglia (Figure 1D,E), while in contrast, DSCLs were characterized by extensive demyelination (Figure 1C) and the presence of amoeboid microglia (Figure 1F). Where MRI classification and histological characterization were incompatible, these cases were excluded from the study, (*n* = 6).

### 2.2. RNA-Sequencing Is Not Compatible with Immuno-LCM-ed Post-Mortem Tissue in the CFAS Cohort

Post-RNA-seq quality control (QC) of the WM samples confirmed the sequencing (single ended, 101 bp) for each sample generated read counts between 3.1 and 16.8 × 10^6^. The sequence outputs for all samples were clear of any contaminated reads from the adaptors. All samples reported a partial read failure, ranging between 36 and 45%. The per-base N assessment had six warning flags for samples with the N ratio passing 5% of the total reads (*n* = 6), and the remaining samples (*n* = 6) failed for having an N ratio higher than 20%. A low diversity of nucleotides was seen as the sequenced data of all samples were dominated by reads that were almost all G and C bases. In addition, the sequence output of all samples had a poor report for the target sequences with a small number of unique reads ranging between 17.7 and 33.3%; the samples had a high level of duplication and were considered over-representative for having a sequence that made up more than 0.1% of the total (Table 1). Following the QC failure, no further interrogation of the data was performed. The RNA-seq datasets are freely available at the Gene Expression Omnibus (GEO) public database (accession code GSE260619).

### 2.3. Microarray Transcriptomic Profiling Is Compatible with Immuno-LCM in the CFAS Cohort

**Figure 2:**
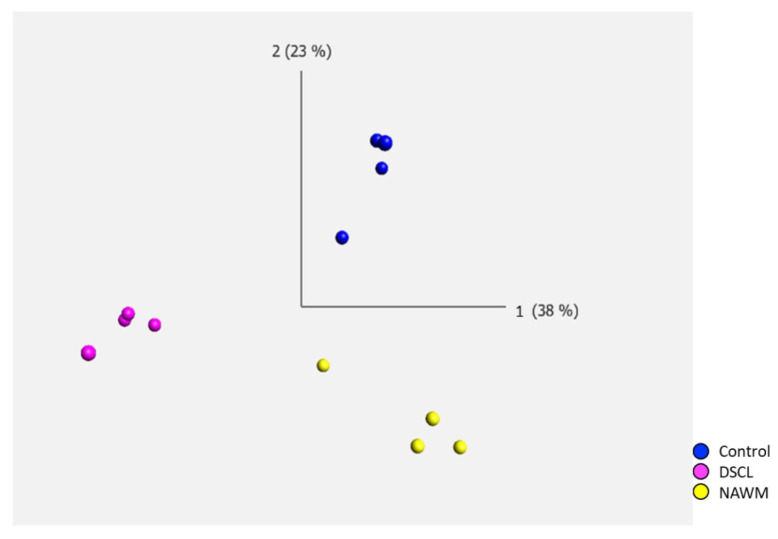
Principal component analysis (PCA) of the microglial transcriptome in ageing white matter. There was a clear separation of the differentially expressed genes between the three white matter groups, while the clustering of cases was similar within groups. Control (*n* = 4); normal-appearing white matter (NAWM, *n* = 4); deep subcortical lesion (DSCL, *n* = 4).

Post-microarray QC were completed on all samples including checks for poly-A RNA labelling controls, hybridization controls, positive versus negative area under the curve (AUC) and signal intensity. No outliers were identified, and all samples underwent subsequent bioinformatic analysis. Principal component analysis (PCA) of the transcriptomic profile of microglia in ageing white matter demonstrated distinct separation between the three WM groups (control, NAWM and DSCL), demonstrating gene expression differences between the groups while displaying genetic similarities within them (Figure 2).

181 significantly differentially expressed genes (DEG) were identified in DSCLs compared to control WM from non-lesional cases (93 upregulated, 88 downregulated), 299 DEG were identified in DSCLs compared to NAWM (97 upregulated, 202 downregulated), and 374 DEG were identified in NAWM compared to control WM from non-lesional cases (209 upregulated, 138 downregulated). The microarray datasets are freely available at the Gene Expression Omnibus (GEO) public database (accession code GSE260815).

KEGG pathway analysis identified no significant dysregulated pathways between-group comparisons, where all Benjamini-corrected *p*-values were >0.05. However, following gene annotated clustering, significant dysregulation of functional groups in DSCLs versus control WM from non-lesional cases were identified, including haptoglobin binding (*p* = 0.017), haptoglobin–haemoglobin complex (*p* = 0.024), TBC domain family member (*p* = 6.3 × 10^−6^) and Rab-GTPase (*p* = 4.3 × 10^−3^) (Table 2). A significant dysregulation of functional groups associated with protein deubiquitination (*p* = 1.3 × 10^−4^) and ubiquitin-dependent protein catabolic processes (*p* = 1.3 × 10^−4^) were identified in NAWM from lesional cases compared to control WM from non-lesional cases (Table 3). In addition, the scavenger receptor class B, member 1 gene (*SRB1*) was significantly upregulated in NAWM compared to control WM (FC + 9.13, *p* = 0.0486). No significant functional groups were detected in DSCLs compared to NAWM.

### 2.4. Validation of Candidate Gene Expression

**Figure 3:**
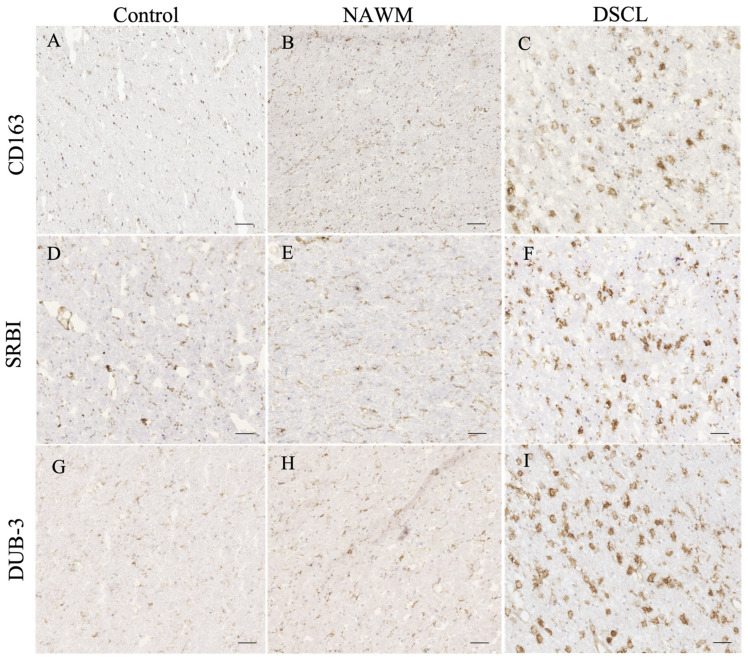
Histological validation of proteins encoded by candidate genes identified from the microarray analysis. The immunoreactive profile of (**A**–**C**) CD163, (**D**–**F**) scavenger receptor class B, member 1 (SRBI), and (**G**–**I**) deubiquitinating enzyme 3 (Dub3) in control, normal-appearing white matter (NAWM) and deep subcortical lesions (DSCLs) indicated expression of the proteins encoded by candidate genes by cells morphologically resembling microglia. Scale bar represents 250 µm.

Extensive immunoreactivity of CD163, scavenger receptor class B, member 1 (SRBI) and deubiquitinating enzyme 3 (Dub3) was a prominent feature of DSCLs compared to both NAWM from lesional cases and control WM from non-lesional cases. Within the DSCL, CD163, SRBI and Dub3 immunolabeled cells morphologically resembling microglia with an amoeboid morphology, while in the control WM and NAWM, the immunoreactivity was associated with the cell body and processes of cells morphologically resembling ramified microglia (Figure 3).

Quantification of the expression of SRBI and Dub3 identified significant differences in the immunoreactive profile between DSCLs and control WM groups; *p* = 0.027 and *p* = 0.018, respectively. However, there was no significant difference in the immunoreactive profile of CD163 across the three groups, *p* > 0.05: Table 4.

## 3. Discussion

While age-associated WMLs are of clinical importance as their accumulation is associated with cognitive decline [12], they remain understudied in the field and the role of microglia in DSCL formation or spread is unknown. Hence, the current study aimed to assess the microglial transcriptomic profile in DSCL, to elucidate their role in lesion pathology. We demonstrate that in the established CFAS neuropathology cohort, microarray analysis but not RNAseq is compatible with the transcriptomic profiling of immuno-LCM-ed post-mortem material. Furthermore, the transcriptomic data reveals complex roles for microglia within DSCLs in response to BBB dysfunction whereby microglia upregulate expression of CD163 and adapt a neuroprotective anti-inflammatory phenotype.

RNA-seq relies on the creation of a cDNA library, followed by sequencing of short fragments, providing reads for specific genes [13,14]. In contrast microarray transcriptomic profiling is a hybridization-based technique, where cDNA fragments are labelled, and hybridized onto the gene chip and gene expression is directly proportional to the signal intensity [15]. While recent studies have shown immuno-LCM is compatible with RNAseq [16], this protocol requires high-quality RNA to enable transcriptome acquisition, which is more readily obtained from snap-frozen surgical or animal tissue than post-mortem neuropathology cohorts. In addition to post-mortem delay-associated RNA degradation, the duration of the immunolabelling and microdissection stages of the protocol further negatively impact RNA integrity [17], which may also have contributed to the failed RNAseq QC in the current study. In contrast, microarray analysis successfully generated robust data, which supports previous findings that the use of microarrays in conjunction with mmune-LCM-ed material is a robust protocol for the molecular analysis of specific CNS cell populations [18]. However, it should be acknowledged that future research is required to establish if the data presented here is a feature of the CFAS or whether it is also applicable to other established neuropathology cohorts.

The present study identified significant upregulation of microglial transcripts associated with haptoglobin (Hp) binding and haptoglobin–haemoglobin binding (Hb–Hp) in DSCL. The irreversible form of Hb–Hp binding has an antioxidant role [19], and forms following dysfunction of the BBB [20]. Disruption of cerebral endothelial integrity and the subsequent release of Hb following the extravasation of red blood cells (RBC) results in the deposition of iron, oxidative stress and cell damage. Previous histological characterization of age-associated WMLs has demonstrated BBB dysfunction and oxidative stress are common features of DSCLs [8,10]. Increased expression of the haemoglobin α and β subunits by glia and neurons has been reported in a range of neurodegenerative diseases, including multiple sclerosis (MS) [21] and intracerebral haemorrhage (ICH) following cerebrovascular damage [22]. In support of the transcriptomic data, the current study demonstrates an increase in the immunoreactive profile of CD163, a macrophage scavenger receptor that rapidly recognizes and degrades the Hb–Hp complex and is a M2-associated microglial marker [23]. Recent microarray analysis of peripheral blood samples from mild cognitive impairment and cognitively normal subjects has also identified *CD163* as a critical gene related to WMLs [24]. We hypothesize that in response to RBC extravasation due to BBB dysfunction in DSCL, microglia upregulate expression of CD163 and adapt a neuroprotective anti-inflammatory phenotype.

While the NAWM from lesional brains and control WM from non-lesional cases both have a similar radiological presentation, their microglial transcriptomic profiles are significantly different. Microarray analysis identified dysregulation of genes associated with protein deubiquitination, including the ubiquitin-specific peptidase 17-like family member (*USP17L*) subfamily, which removes conjugated ubiquitin from target proteins [25]. The downregulation of a range of deubiquitinating enzymes (DUBs) has been reported in several neurodegenerative diseases impacting inflammation, cell motility, carcinogenesis, neuronal autophagy and the response to oxidative stress [26,27,28,29]. Dysregulation of protein ubiquitination and deubiquitination in the NAWM infers a change of microglial function in the regions surrounding DSCL, which may contribute to the progression of lesion pathology.

In contrast to studies that have suggested that WMLs are symmetrical [30], the current findings provide evidence that this is not always the case and highlight the importance of performing histological evaluation of MRI-guided samples of the contralateral hemisphere. While transcriptomic profiling can potentially identify novel and biologically relevant mechanisms associated with disease pathogenesis, it should be acknowledged that microarrays only analyze transcripts that are fully annotated and have a lower sensitivity than RNAseq [31]. Current recommendations suggest transcriptomic profiling should be conducted on a minimum of twelve samples per group [32]; therefore, a further limitation of the current study is the low number of cases assessed. Nonetheless the significantly differentially expressed candidates identified in the current study warrant further investigation, as modulation of the neuroinflammatory response may be a therapeutic target to preserve brain function in the ageing brain.

## 4. Materials and Methods

**Figure 4:**
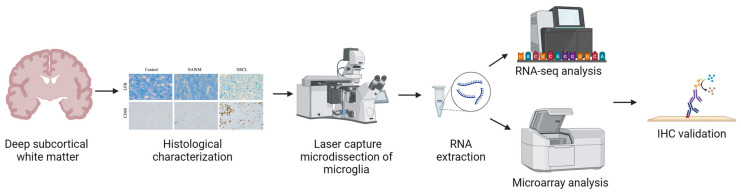
Overview of study design. Deep subcortical white matter blocks were histologically characterized to confirm either non-lesional control or lesional white matter had been sampled. CD68^+^ microglia were isolated using immuno-laser capture microdissection and the transcriptomic profile was assessed using RNA-seq and microarray analysis. Immunohistochemistry (IHC) was performed to confirm expression and cellular localization of proteins encoded by a panel of candidate genes. Created with BioRender.com.

A workflow of the study design is outlined in Figure 4.

### 4.1. Case Selection and Histological Characterization

Frozen post-mortem deep-subcortical white matter blocks were obtained from the Cognitive Function and Ageing Study (CFAS) neuropathology brain bank in accordance with Research Ethics Committee approval (REC No: 15/SW/0246). Initially, MRI analysis of the formalin-fixed hemisphere was used to guide case selection and sampling of the contralateral frozen hemisphere. The modified Schelten’s semi-quantitative rating system was used to identify the control (*n* = 4) and lesional cases (*n* = 4), where control WM cases had a score of 0, and DSCLs had a score of 5. Matched DSCLs (*n* = 4) and NAWM (*n* = 4) blocks were obtained from the lesional cases. The mean age at death of the control samples was 81.8 years, (SD 8.2 years, range 73–92 years), and the DSCL samples was 89.7 years, (SD 6.5 years, range 74–96 years). The post-mortem interval in the control group ranged between 6 and 75 h, the DSCL group ranged between 15 and 79 h.

Previous studies have shown that DSCLs are characterized by high levels of CD68^+^ microglia with an amoeboid phenotype [10]; therefore, to confirm that DSCLs had been sampled, expression of CD68 (Mouse monoclonal, IgG 1:100, Dako, Ely, UK) was assessed using a standard horseradish peroxidase-conjugated avidin-biotin complex (ABC-HRP) immunohistochemistry approach (Vectastain Elite kit; Vector Laboratories, Peterborough, UK) with 3,3′-diaminobenzidine (DAB) (Vector Laboratories UK) as the substrate.

### 4.2. Laser Capture Microdissection (LCM) of Microglia

CD68 immunopositive microglia were isolated from the deep-subcortical white matter blocks (12 white matter blocks: control *n* = 4; NAWM *n* = 4; DSCLs *n* = 4) using a modified rapid immuno-laser capture microdissection (LCM) protocol using the Arcturus PixCell II LCM system (Thermoscientifc, Runcorn, UK). Approximately 2500 CD68^+^ cells were isolated from each case using LCM and total RNA was extracted using the Arcturus PicoPure RNA isolation kit according to the manufacturer’s protocol (Thermoscientifc, Runcorn, UK).

### 4.3. RNA-Sequencing

RNA-sequencing of 12 samples (control *n* = 4; NAWM *n* = 4; DSCLs *n* = 4) was performed at the Sheffield Diagnostic Genetics Service, Sheffield Children’s Hospital NHS Foundation Trust. RNA was prepared for sequencing using the NEBNext^®^ Single Cell/Low Input RNA Library Prep Kit (Illumina, San Diego, CA, USA) according to the manufacturer’s protocol. Briefly, 8 μL of extracted total RNA was reversed transcribed to cDNA and amplified following 20 cycles of amplifying PCR (initial denaturation at 98 °C for 45 s, the product was amplified at 98 °C for 10 s, 62 °C for 15 s, 72 °C for 3 min and then 72 °C for 5 min). Following fragmentation, samples underwent adapter ligation and indexing to create cDNA libraries, permitting recognition of each sample once pooled. Each cDNA library product underwent amplification following 9 cycles of amplifying PCR (initial denaturation at 98 °C for 30 s, then the product was amplified at 98 °C for 10 s, 65 °C for 75 s and then 65 °C for 5 min) and pooled. Pooled cDNA libraries were run in duplicate lanes of a flow cell to maximize the number of mapped reads (100 bp single read) and sequenced on an Illumina HiSeq 2500 platform using the Illumina TruSeq SBS Kit, San Diego, CA, USA).

#### RNA-Seq Data Analysis

Galaxy web platform, at usegalaxy.org version 1.9, was accessed on 12 January 2021 and used for sample quality assessment, alignment, quantification and differential expression analysis. Concatenate Datasets tool in Galaxy was used to combine the two fastq files for each sample and subsequent quality checks were carried out using the MultiQC tool that visually presents a summary comparison of the output from numerous data across all samples. Using the HISAT2 tool, sample reads from the concatenated fastq files were mapped and aligned to the built-in reference human genome (Hg38). Compact Idiosyncratic Gapped Alignment Report (CIGAR) was generated to pair the given sequences with their assigned position on the reference genome. The QC statistics for aligned reads were calculated using samtools, enabling the flagging of any unmapped, unpaired or duplicated reads.

### 4.4. Microarray

RNA extracted from CD68^+^ve LCM cells of 12 samples (control *n* = 4; NAWM *n* = 4; DSCLs *n* = 4) was prepared for microarray analysis using the GeneChip 3′ IVT Pico kit (Applied Biosystems, ThermoFisher, UK). In brief, 10 ng RNA was amplified using low-cycle PCR followed by amplification using T7 in vitro transcription. The cRNA was converted to biotinylated sense-strand DNA hybridization targets. Approximately 5.5 μg amplified cDNA was fragmented, labelled and hybridized to Clariom S Array chips for 16 h at 45 °C in a rotating oven at 60 rpm. Using the Fluidics Station 400 (Affymetrix^®^, High Wycombe, UK) and GeneChip Operating System (Affymetrix^®^, High Wycombe, UK), a series of washing steps followed to remove any unbound DNA, before each microarray was stained and scanned using the GeneChip 3000 scanner (Affymetrix^®^, High Wycombe, UK).

#### Microarray Data Analysis

Affymetrix Expression Console software version 1.4.1.46 (Affymetrix^®^, High Wycombe, UK) was employed to quality control the data and principal component analysis (PCA) in Qlucore Omics Explorer software version 3.9 (Qlucore, Lund, Sweden) was used to visually inspect the data and identify sample outliers. Transcriptome Analysis Console (TAC) software version 4.1.1 (Affymetrix^®^, High Wycombe, UK), was used to analyze and compare the gene expression profile of the 3 groups. Genes were considered significantly differentially expressed if they had a minimum fold change (FC) ≥ ±1.2 and *p* ≤ 0.05. The comparisons of the microglial transcriptomic profiles in each WM group were analyzed using the Database for Annotation Visualization and Integrated Discovery (DAVID) software version 6.8) [33]. Each dataset from the 3 comparisons was uploaded to DAVID to identify significant KEGG (Kyoto Encyclopedia of Genes and Genomes) pathways. The clustering tool on DAVID was used at the highest stringency settings to obtain high specificity and minimize the rate of false-positives, and to enable gene annotated clustering of groups according to their related function, pathway or interactions.

### 4.5. Immunohistochemical Validation of Protein Changes

To confirm expression and cellular localization of proteins encoded by a panel of candidate genes, standard ABC-HRP immunohistochemistry was carried out with DAB as substrate (Vectastain Elite kit; Vector Laboratories UK) on the control (*n* = 4), NAWM (*n* = 4) and DSCLs (*n* = 4) white matter blocks used in the transcriptomic studies. The primary antibodies and experimental conditions used are shown in Table 5. For image acquisition, both the Nikon microscope and NIS-Elements Imaging Software version 4.2 (Nikon, Surbiton, UK, Kingston Upon Thames) were used. Analysis^D (Nikon UK, Kingston Upon Thames) was used to quantify the percentage of immunoreactivity across 5 images of the deep subcortical white matter area for each case.

#### Statistical Analysis

The relationship between the different markers within the different patient groups was determined using a non-parametric Kruskal–Wallis test with the Bonferroni correction test using SPSS software version 26.0 (SPSS Inc., Chicago, IL, USA).

## Figures and Tables

**Table 1:** Quality control analysis of RNA-seq data using the Galaxy platform. Samples which passed or failed quality controls are indicated in blue or orange, respectively; mapped sequences (M seq), per base GC content, sequence duplication and over-represented sequences of 12 white matter cases (control *n* = 4; NAWM *n* = 4; DSCLs *n* = 4).

Sample ID	Length	M Seq (×10^6^)	% Fail	% GC	% Dup	% Over-Representative
Sample 1_fastq	101 bp	3.1	45%	87%	76.5%	23.5%
Sample 2_fastq	101 bp	6	36%	87%	77.7%	23.5%
Sample 3_fastq	101 bp	6.9	36%	90%	82.3%	25.0%
Sample 4_fastq	101 bp	7.8	36%	88%	80.0%	25.0%
Sample 5_fastq	101 bp	16.5	45%	83%	67.3%	21.5%
Sample 6_fastq	101 bp	6.3	36%	89%	80.9%	25.0%
Sample 7_fastq	101 bp	6.1	45%	90%	81.7%	23.5%
Sample 8_fastq	101 bp	9.5	45%	80%	66.8%	19.7%
Sample 9_fastq	101 bp	16.8	45%	83%	78.8%	21.5%
Sample 10_fastq	101 bp	8.4	36%	89%	80.1%	19.7%
Sample 11_fastq	101 bp	9.6	36%	89%	81.2%	25.0%
Sample 12_fastq	101 bp	8.9	45%	90%	80.8%	19.7%

**Table 2:** Functional group analysis of the microglial profile in deep subcortical lesions (*n* = 4) versus control white matter from non-lesional cases (*n* = 4). Significant differentially expressed genes were analyzed using the Database for Annotation Visualization and Integrated Discovery (DAVID) software.

Annotated Cluster	Enrichment Score	Gene Count	Benjamini *p*-Value	Directional Change	Differentially Expressed Genes
Haptoglobin binding	2.5	3	1.7 × 10^−2^	Up	Haemoglobin-α1 (*HBA1*) Haemoglobin-α2 (*HBA2*) Haemoglobin-β (*HBB*)
Haptoglobin–haemoglobin complex		3	2.4 × 10^−2^		
TBC	2.66	7	6.3 × 10^−6^	Down	TBC1 domain family member: (*TBC1D3B*, *TBC1D3E*, *TBC1D3F*, *TBC1D3G*, *TBC1D3H*, *TBC1D3I*, *TBC1D3L*)
Rab-GTPase-TBC domain			4.3 × 10^−3^		

**Table 3:** Functional group analysis of the microglial profile in normal-appearing white matter from lesional cases (*n* = 4) versus control WM from non-lesional cases (*n* = 4). Significant differentially expressed genes were analyzed using the Database for Annotation Visualization and Integrated Discovery (DAVID) software.

Annotated Cluster	Enrichment Score	Gene Count	Benjamini *p*-Value	Directional Change	Differentially Expressed Genes
Protein deubiquitination	5.14	9	1.3 × 10^−4^	Down	Ubiquitin specific peptidase 17-like family member: *USP17L5*, *USP17L19*, *USP17L24*, *USP17L25*, *USP17L26*, *USP17L27*, *USP17L28*, *USP17L29*, *USP17L30*, Ring finger protein 8 (*RNF8*) Toll interaction protein (*TOLLIP*)
Ubiquitin-dependent protein catabolic process		11	1.3 × 10^−4^		

**Table 4:** Quantitative assessment of CD163, SRBI and Dub3 protein expression in age-associated deep subcortical white matter. The mean percentage area (and range) of immunoreactivity of each of the markers is shown in control, normal-appearing white matter (NAWM) and deep subcortical lesions (DSCLs).

WM Group	Control (*n* = 4)	NAWM (*n* = 4)	DSCLs (*n* = 4)
CD163	0.47 (0.29–0.85)	1.41 (0.43–3.24)	4.07 (2.12–6.41)
SRBI	0.46 (0.22–0.70)	0.83 (0.74–0.93)	7.02 (1.85–15.09) *
Dub3	0.25 (0.20–0.28)	0.44 (0.30–0.66)	4.44 (1.09–7.89) *

* *p* < 0.05 (Kruskal–Wallis with Bonferroni correction).

**Table 5:** Antibody source and specificity.

Primary Antibody	Species	Clonality	Isotype	Dilution	Supplier
CD163	Rabbit	Monoclonal	IgG	1:500	Abcam, UK
Dub3	Rabbit	Polyclonal	IgG	1:100	Novusbio, UK
SRBI	Rabbit	Monoclonal	IgG	1:500	Abcam, UK

## Data Availability

The RNA-seq and microarray datasets are freely available at the Gene Expression Omnibus (GEO) public database (accession code GSE260619 and GSE260815, respectively).
